# Employing open/hidden administration in psychotherapy research: A randomized-controlled trial of expressive writing

**DOI:** 10.1371/journal.pone.0187400

**Published:** 2017-11-27

**Authors:** Theresa Tondorf, Lisa-Katrin Kaufmann, Alexander Degel, Cosima Locher, Johanna Birkhäuer, Heike Gerger, Ulrike Ehlert, Jens Gaab

**Affiliations:** 1 Clinical Psychology and Psychotherapy, Department of Psychology, University of Basel, Basel, Switzerland; 2 Clinical Psychology and Psychotherapy, Institute of Psychology, University of Zurich, Zurich, Switzerland; Public Library of Science, UNITED STATES

## Abstract

Psychotherapy has been shown to be effective, but efforts to prove specific effects by placebo-controlled trials have been practically and conceptually hampered. We propose that adopting open/hidden designs from placebo research would offer a possible way to establish specificity in psychotherapy. Therefore, we tested the effects of providing opposing treatment rationales in an online expressive writing intervention on affect in healthy subjects. Results indicate that it was possible to conduct the expressive writing intervention both covertly and openly, but that participants in the hidden administration condition did not fully benefit from the otherwise effective expressive writing intervention in the long-run. Effect sizes between open and hidden administration groups were comparable to pre-post effect sizes of the intervention. While this finding is important for the understanding of psychotherapy's effects per se, it also proves that alternative research approaches to establish specificity are feasible and informative in psychotherapy research.

**Trial registration:** German Clinical Trials Register DRKS00009428

## Introduction

There is consensus that psychotherapy is effective, yet identification of accountable factors or processes is subject to controversy [1]. Whereas in biomedical research the position that "preservation of sound judgment both in the laboratory and in the clinic requires the use of the double blind technique [2] is considered gold standard, this approach is not fully applicable in psychotherapy research. Although it needs to be noted that even in biomedical research not everything that glitters is gold [3], the principle of indistinguishability between comparators is fundamentally hampered in psychotherapy trials both practically and conceptually [4]. So far, two main approaches have been employed to handle this problem and to establish specificity in psychotherapy research, both with problematic consequences in their own right ([5], p. 213ff).

First, since it is old lore that "to show that a specific form of psychotherapy (…) produces results not attributable to the non-specific placebo effect it is not sufficient to compare its results with (…) no treatment" (cited from [6], p. 300) psychotherapy is to be compared to psychological placebo to establish specificity of the treatment [7]. Placebo conditions often run under various denominators, such as supportive therapy, nondirective therapy, common factor control, credible attention placebo, and modest contact, but should be and are considered as placebo as they are lacking treatment constituents deemed specific. This seemingly adopts the gold standard of biomedical research and thus is considered good enough to be gentled as "Research-supported psychological treatment" by the American Psychological Association [8]. However, there is evidence that differences between psychotherapies and psychological placebo conditions are a function of the credibility of the placebo control [9, 10] as well as the extent of structural equivalence between comparators [11] and that controlling for researchers' allegiance towards a particular intervention abolishes observed differences (e.g. [12]). Second, specificity has been sought through direct comparisons of psychotherapies, which usually differ in their characteristic, but not in their incidental treatment constituents [13] and through dismantling or additive component studies, in which presumable characteristic treatment constituents are either omitted or added. However, meta-analyses on respective trials do not substantiate the existence of statistically and/or clinically significant differential effects per se [14, 15, 16, 17, 18], besides those are related to researchers' allegiance [19].

In conclusion, the utility of randomized placebo-controlled trials in clinical research "to sort therapeutic wheat from chaff" (cited from [20], p. 1502) is not unequivocally warranted in psychotherapy. Given that placebo conditions in psychotherapy cannot be blinded, the main principle of randomized placebo-controlled trials, i.e. to employ different interventions with the same rationale–or put otherwise: to control the incidental, while manipulating the characteristic treatment constituents–is not applicable. However, the strive for specificity–understood here as the identification of characteristic treatment constituents outperforming incidental ones–is important regardless of both the understanding of psychotherapy or of the respective characteristic treatment constituent under investigation.

Here, placebo research designs could come to aid. Using a topsy-turvy approach with regard to randomized placebo-controlled trials, the aim is to control for characteristic, while manipulating incidental treatment constituents. A stringent implementation of this approach is the open versus hidden design, where an otherwise identical intervention is given to subjects either knowing or not knowing when or if the drug will be administered or withdrawn. Differences between conditions are then understood as placebo-like effects, since the intervention itself is not a placebo (e.g. [21]). This trial design has the merits of being able to specify effects of assumingly incidental treatment constituents, e.g. therapeutic meaning [22], from those of characteristic treatment constituents without the use of a placebo condition and thus has successfully been used in non-pharmacological interventions (e.g. [23, 24]). However, it has not been tested in the realm of psychotherapy interventions. Employing an online expressive writing intervention, we tested the feasibility of an open/hidden paradigm in psychotherapy and whether the effects of the assumingly characteristic treatment constituent of expressing emotions about a traumatic experience are influenced by the assumingly incidental treatment constituent of providing a therapeutic rationale, i.e. a meaning [25].

## Methods

### Design

We conducted a randomized controlled trial with three experimental conditions in healthy subjects (see S1 File for trial and manuscript details). Two groups underwent a standardized online intervention, whereas the control group did not receive an intervention. While both intervention groups received the same online intervention, they were given different treatment rationales for the intervention: Participants in the causality group were told that the intervention will have beneficial effects on mood in the long-run, participants in the reversed causality group were instructed that mood will influence how they will perform in the intervention (see S2 File for details).

The intervention consisted of an online version of the expressive writing paradigm [26], due to its feasibility and proven efficacy to reduce distress in healthy student and clinical populations (e.g. [27, 28]. It needs to be noted that expressive writing leads to short-term deterioration of affect, followed by long-term increase in well-being [29]. Subjects in both intervention groups wrote about their most traumatic experience on three consecutive days for 20 minutes on each day.

### Procedures

In total, participants were imbedded in the study for 46 days. The study encompassed three intervention days (days 1 to 3) and four assessments (days 1, 4, 10 and 46 or baseline, post intervention, mid-term and long-term follow-up, respectively). On intervention and assessment days participants received an email with an individual access code for a webpage differing in content according to group assignment and study day. For all groups, the website contained the outcome (PANAS, see below). On the intervention days, the website also contained an embedded video for the two intervention groups, showing a professional speaker explaining the rationale of the intervention. While the instruction was equal for both intervention groups in terms of structure and format, the treatment rationale, i.e. proposed meaning, differed in content between these groups (S2 File). For the control group, this website contained the outcome only. On intervention days, participants of the intervention groups completed the outcome before and after the expressive writing intervention, while the control group completed the outcome only once on each of the intervention days. The intervention, the different instructions as well as the assessments were conducted online in order to control for patient-therapist interaction. Participants were not informed about the existence of and the difference between the three groups. Subjects were debriefed after study participation. Participants were provided with written information about the study and written informed consent to participate in this study was obtained online and stored digitally. The protocol and consent procedure was approved by the institutional review board of the Faculty of Psychology of the University of Basel (S3 File, S4 File). The trial was not registered in a WHO approved registry before enrolment, because the trial initially was not considered to qualify as a clinical trial and comparable trials have not been registered as clinical trials (e.g. [27, 28, 30, 31]). However, the study was retrospectively registered at the German Clinical Trials Register (S5 File). The authors confirm that all ongoing and related trials for this intervention are registered. There were no changes to methods after trial commencement.

### Measures

Possible effects of the experimental conditions were repeatedly assessed for up to 46 days after baseline with the Positive and Negative Affect Schedule (PANAS, [32]), which was *a priori* defined as the primary outcome. The PANAS contains two scales with overall 10 five-point items assessing positive (e.g. "interested", "proud") and negative affect (e.g. "upset", "ashamed"). The internal consistency estimate of reliability for the PANAS scales in the total sample was good (positive affect: Cronbach’s alpha = 0.87, negative affect: Cronbach’s alpha = 0.79). In the intervention groups, linguistic content of written text, subjective rating of the severity of the reported traumatic experiences and the plausibility of their respective treatment rationale were employed to assess the validity of the intervention and experimental manipulation of treatment rationale. The linguistic content of the written text was analyzed with *Linguistic Inquiry and Word Count*, which quantifies words in a given text according to preset categories with a 70–80% hit rate [33]. For the purposes of this study, we used the word count of first person singular personal pronouns (e.g. *I*, *me*, *mine*), of negative and positive emotions (e.g. *sad*, *hate*, *worthless* and *happy*, *pretty*, *good*), of cognitive processes (e.g. *distinguish*, *because*, *know*), of causality (e.g. *argument*, *influence*, *effect)* and of insight (e.g. *recognize*, *conscious*, *decision*). Subjective rating of the severity of the traumatic experiences was assessed with a single sentence 5-point item (i.e. *In general*, *how distressing is the experience you have just written about for you*?) and plausibility of the treatment rationale was operationalized with a 5-point item for each group (causality group: *Writing about a traumatic experience influences my well-being*, reversed causality group: *My well-being influences how I write about a traumatic experience)*. All described measures were assessed online and stored in internal and secured data carriers.

### Subjects

**Fig 1:**
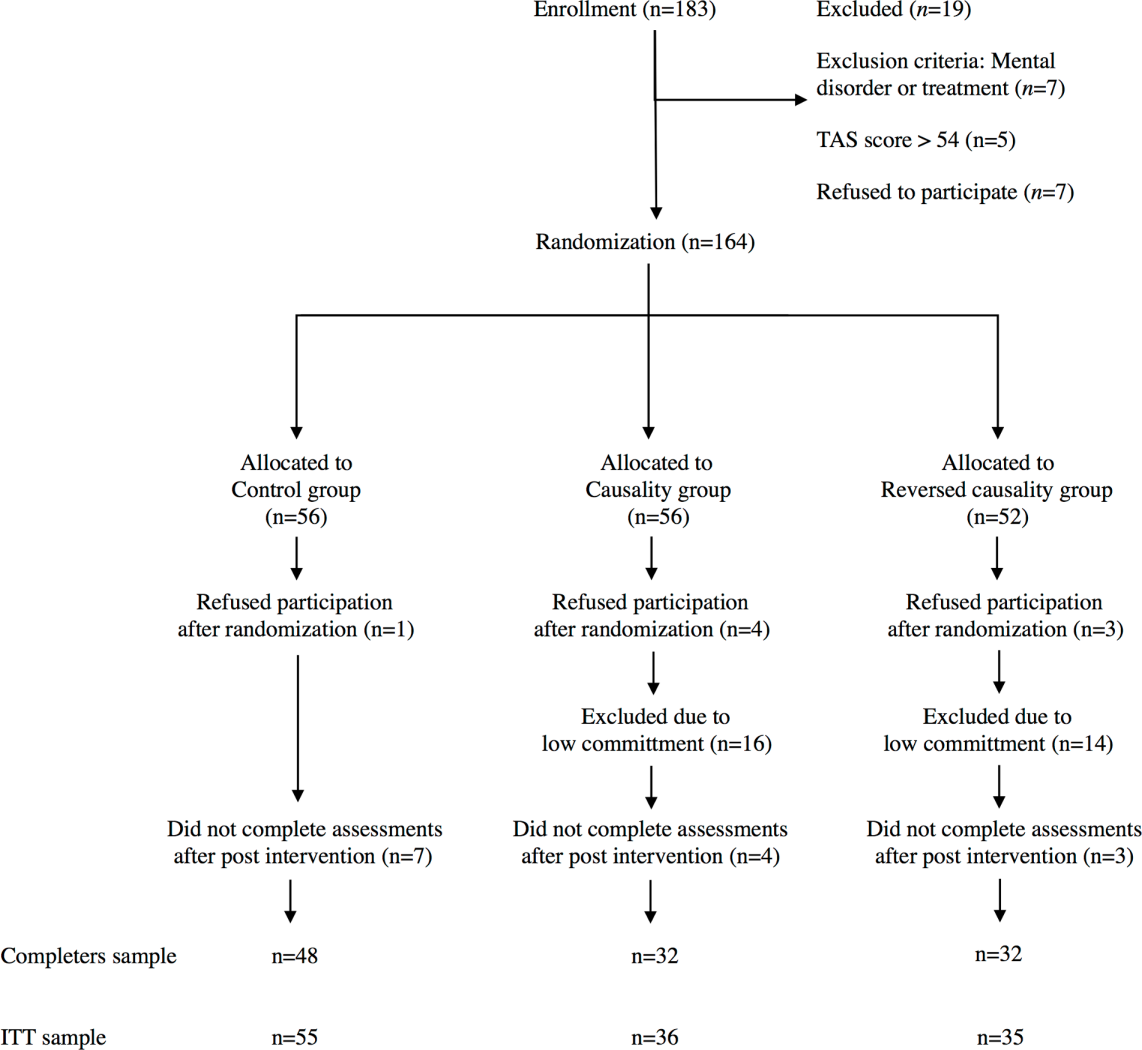
Flow of participants through the study (ITT: Intention to treat, TAS: Toronto Alexithymia Scale).

Subjects were recruited amongst psychology students at two Swiss Universities (University of Zurich and University of Basel) through mailing lists and web postings at both universities. Inclusion criteria were (1) ages of 18 years and older, (2) absence of any mental disorder by self-report, (3) not receiving psychological or psychiatric or medical treatment in the last six month by self-report and (4) a Toronto Alexithymia Scale score below 54 [34], since alexithymia has been shown to influence effects of expressive writing [35]. Of 183 enrolled subjects, 19 did not fulfill inclusion criteria and were therefore excluded. A total of 164 subjects were randomly assigned to the three experimental groups and 112 participants completed the intervention and all assessments. Random allocation sequences were achieved with individual assignment codes in separate envelopes, opened by study personnel after confirmation of eligibility and informed consent. Eight subjects dropped out after randomization and before post-intervention assessment and 30 subjects were excluded due to low commitment in the expressive writing task, defined as writing for less than 15 minutes on intervention days by self-report, so that overall 126 subjects were considered per protocol (control group N = 55, causality group N = 36, reversed causality group N = 35). Furthermore, 14 subjects did not complete all assessments after post-assessment, so that two samples were analyzed (completers N = 112 and ITT N = 126, see Fig 1). Subjects not meeting inclusion criteria as well as those excluded due to low commitment, i.e. not being considered as per protocol, did not differ significantly in any baseline demographic or baseline parameters (all p>0.40). Mean age of participants was 23 years and 8 months (control group 22 years and 9 months; causality group 24 years and 11 months; reversed causality group 23 years and 11 months) and the gender ratio was 86 women and 26 men (control group 37/11; causality group 25/7; reversed causality group 24/8). Overall, only 2 participants (1.8%) kept a regular diary. Participants received study credits for their participation. The flow of participants through the study is shown in Fig 1.

### Data analysis

Based on assumed small to medium effects of our experimental manipulation of treatment rationale on intervention effects, a priori sample size calculation with the statistical software G*Power 3.1 [36] led to an optimal sample size of N = 120 (f = 0.2, 80% power, 5% alpha error, 3 groups, 4 assessments and correlation among repeated measures = 0.3). We assumed that our restrictive exclusion criteria, the population under investigation and the emotionally demanding task would lead to a substantial dropout of at least 30%. We therefore set out to recruit N = 180. SPSS 21 statistical software (SPSS, Chicago, Illinois, USA) for Apple OS X was used for all statistical analyses. Based on previous recommendations, we did not run statistical tests comparing the participants on baseline demographic characteristics and outcome measures (e.g. [37]).

A time by group by scale multivariate analysis of variance with subsequent time by group univariate analysis of variance for single PANAS scales was used to investigate differences between groups regarding treatment effects over time. For significant results between groups, Cohens f (calculated from SPSS partial eta-squared with the program G*Power 3.1: 0.1 = small, 0.25 = medium and 0.45 = large) was used for time by group interaction effects. Within- and between-group effect sizes were calculated using Cohen's d (0.2 = small, 0.5 = medium, 0.8 = large). All results are displayed as mean values and standard deviation unless otherwise indicated. All analyses were performed on per protocol subjects, i.e. with sufficient commitment in the writing task, as we were interested in the effects of different rationales for a given treatment. For this, it is of importance to ensure treatment fidelity in the first place. Otherwise, there would not be any “administration of a treatment” at all and in consequence, no open/hidden administration of a treatment. Clearly, the low-commitment cases were not undergoing the expressive writing intervention and thus any inclusion of them would have violated both our aim to test this as well as the validity of our data. In this regard, it is of importance to note that the number of low-committers is near similar in both intervention groups (Fig 1), pointing towards that the open/hidden administration did not have a systemic effect on commitment. All analyses of on protocol subjects were performed on an intent-to-treat (ITT) basis including randomized protocol subjects with baseline and at least post-intervention values (day 4) for the variable being analyzed (see Fig 1). All data is accessible in S6 File.

## Results

### Characteristics of participants and validation check of intervention

Experimental groups did not differ significantly regarding gender distribution (female/male: causality group: 25/7, reversed causality group: 24/8 and control group: 37/11), baseline PANAS scores (Table 1) and Toronto Alexithymia Scale total score (control group: 38.0 (6.9), causality group: 38.6 (6.3) and reversed causality group: 35.9 (6.0)). Further, intervention groups did not differ substantially in linguistic content of written texts during the three intervention days, but participants in the reversed causality group used more first person singular personal pronouns in comparison to participants in the causality group on the first intervention day (Table 2). The causality and reversed causality groups reported comparable severity of the traumatic experiences on all three days and did not differ substantially in their ratings of the plausibility of treatment rationale (Table 3). The mean number of reported experienced traumatic events or troubling issues during the three intervention days was 1.9 (0.9) (one event: N = 28, two events: N = 17 and three events: N = 19). The three most prevalent topics were: *Death* or *severe illness of family members* (e.g. cancer of father, 25%), *parental relationship problems* (e.g. divorce of parents, 22%) and *personal relationship problems* (e.g. adultery of spouse, 18%).

**Table 1:** PANAS scores before and after the expressive writing intervention during the three intervention days and for the mid- and long-term follow-up assessments.

Group	PANAS scale	Day 1		Day 2		Day 3		Day 4	Day 10	Day 46
		before	after	before	after	before	after			
Control group	Positive affect	28.9 (1.0)	-	28.8 (1.0)	-	28.7 (1.1)	-	28.9 (1.0)	27.8 (1.0)	28.7 (1.0)
	Negative affect	12.4 (0.5)	-	12.0 (0.5)	-	12.9 (0.6)	-	12.3 (0.5)	12.6 (0.5)	13.1 (0.5)
Causality group	Positive affect	28.3 (1.2)	25.9 (1.3)	29.0 (1.2)	28.3 (1.4)	26.1 (1.3)	26.8 (1.4)	27.4 (1.2)	28.2 (1.2)	32.0 (1.2)
	Negative affect	12.8 (0.7)	17.6 (1.1)	12.8 (0.6)	14.2 (1.0)	12.0 (0.7)	13.3 (0.8)	12.8 (0.6)	12.7 (0.6)	11.4 (0.6)
Reversed causality group	Positive affect	29.8 (1.2)	27.2 (1.3)	29.3 (1.2)	26.2 (1.4)	26.7 (1.3)	27.1 (1.4)	29.1 (1.2)	29.3 (2.2)	32.0 (1.2)
	Negative affect	12.1 (0.7)	15.5 (1.3)	12.2 (1.2)	16.1 (1.4)	12.7 (1.3)	14.8 (1.4)	11.5 (0.6)	12.0 (0.6)	12.9 (0.6)

**Table 2:** Comparison of linguistic content of written text between intervention groups.

Mean word count in LCWT domain	Day 1		Day 2		Day 3	
	Causality group	Reversed causality group	Causality group	Reversed causality group	Causality group	Reversed causality group
First person singular personal pronouns	9.0 (2.5)	11.2 (2.6)	9.9 (2.4)	9.6 (2.3)	9.6 (3.2)	10.5 (2.9)
Positive emotions	3.6 (1.4)	3.7 (1.4)	3.1 (1.1)	3.6 (1.4)	3.6 (1.2)	3.6 (1.2)
Negative emotions	2.8 (1.1)	2.7 (1.1)	3.0 (1.2)	2.7 (1.0)	2.7 (1.0)	2.7 (1.1)
Cognitive processes	10.9 (2.3)	11.8 (2.2)	12.1 (2.7)	12.4 (2.5)	11.9 (1.8)	12.3 (2.4)
Causality	1.5 (0.6)	1.6 (0.7)	1.7 (0.8)	1.8 (0.9)	1.3 (0.7)	1.7 (0.8)
Insight	3.3 (0.9)	3.9 (1.4)	3.6 (1.2)	3.7 (1.6)	3.3 (0.9)	3.7 (1.2)

**Table 3:** Validation check of intervention: Comparison of severity of traumatic experience and plausibility of study rationale between intervention groups.

Group	Severity of the traumatic experiences°			Plausibility of study rationale°
	Day 1	Day 2	Day 3	
Causality group	3.7 (1.0)	3.3 (0.9)	3.5 (0.9)	4.1 (0.9)
Reversed causality group	3.7 (1.1)	3.4 (1.1)	3.2 (1.1)	4.2 (0.9)

°Range 1–5, higher values indicate higher agreement

The expressive writing intervention led to significant decreases in positive as well as significant increases in negative affect (pre-post time effect: F*(*2/61) = 15.7; p<0.001, f = 0.72, Table 3), which both gradually attenuated over time (intervention day by pre-post interaction effect F(4/248) = 4.5, p = 0.002, f = 0.27). Both intervention groups did not differ in their change of affect following the intervention (pre-post by group interaction effect: F(2/61) = 0.4, p = 0.66). Since participants in the control group were not subjected to the expressive writing intervention and thus a pre- and post-assessment on each day was not feasible, single assessments of the PANAS on days 1 to 3 were performed and compared with pre-intervention scores of the two intervention groups using a repeated multivariate analysis (Table 1). The PANAS scores in the control group did not change over time (F(4/188) = 0.9, p = 0.45).

### Effects of the experimental manipulation on per protocol subjects

To test for possible effect of our experimental manipulation of the treatment rationale in the expressive writing intervention, short-, mid- and long-term effects were examined using repeated multivariate analyses between all three groups.

**Fig 2:**
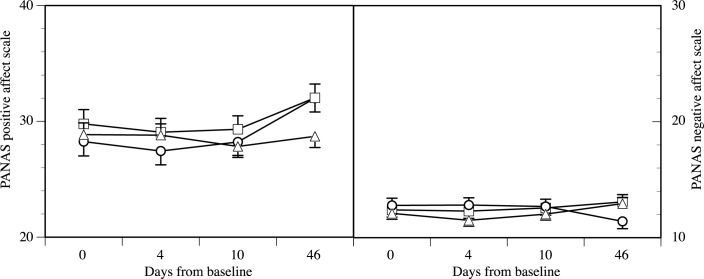
Changes in PANAS positive and negative affect scale scores over time in all experimental groups (Causality group: ○, Reversed causality group: □, Control group: △). Please note the differences in scaling between positive and negative affects scores and that the minimum score for both scales is 10.

To assess short-term effects, differences in PANAS scores between day 1 (pre-assessment) and day 4 (1 day post-intervention) were calculated for the three groups. There was no change in PANAS scores over time (time effect: F(2/108) = 0.5, p = 0.60) or between groups (time by group interaction effect: F(4/218) = 0.2, p = .94). To assess mid-term effects, group differences between day 1 to day 10 (7 days post-intervention) were calculated. Again, there were no changes in PANAS scores over time (time effect: F(2/108) = 0.2, p = 0.80) or between groups (time by group interaction effect: (F(4/218) = 0.1, p = 98). However, regarding long-term effects, differences in PANAS scores between day 1 to day 46 were significant (time effect: F(2/108) = 3.3, p = 0.04, f = 0.25) and groups differed in the long-term changes of positive and negative affect (time by group interaction effect: F(4/218) = 2.9, p = 0.02, f = 0.23). Significant group differences were found for both positive (F(2/108) = 3.6, p = 0.03, f = 0.25) and negative affect (F(2/108) = 4.2, p = 0.02, f = 0.27; see Fig 2). Post-hoc univariate comparisons on PANAS scores on day 46 indicated that with regard to positive affect, interventions groups did not differ significantly from each other, but both differed significantly from the control group (causality group vs. control group: F(1/78) = 4.2, p = 0.04, f = 0.23; reversed causality group vs. control group: F(1/78) = 4.6, p = 0.04, f = 0.24; causality group vs. reversed causality group: F(1/62) = 0.02, p = 0.98). With regard to negative affect the reversed causality group did not differ from the control group, but both groups differed from the causality group (causality group vs. control group: F(1/78) = 4.6, p = 0.04, f = 0.24; reversed causality group vs. control group: F(1/78) = 0.03, p = 0.88; causality group vs. reversed causality group: F(1/62) = 4.0, p = 0.05, f = 0.25). Standardized mean differences for significant effects were of small to medium size for positive affect (standardized between-groups differences at follow-up: causality group versus control group, d = 0.49; reversed causality group versus control group, d = 0.50; standardized within-group baseline to follow-up differences: causality group, d = 0.55; reversed causality group, d = 0.37;control group, d = 0.01) and for negative affect (between groups at follow-up: causality group versus control group, d = 0.57; causality group versus reversed causality group, d = 0.64; standardized within group baseline to follow-up differences: causality group, d = 0.44; reversed causality group, d = -0.22;control group, d = -0.19).

### Effects of the experimental manipulation on ITT sample

Results of analyses on participants who were on treatment and completed at least the day 4 (post-intervention) assessment did not differ from the completers sample with regard to sample characteristics of participants, validation check of intervention as well as the magnitude of the short- and mid-term changes of PANAS scores between groups. Also, the results for the long-term data of ITT sample was comparable to those of the completers sample, with minor changes of type one error for the time effect for PANAS scores and group by time interaction effect for positive affect.

Repeated multivariate analysis of group differences in PANAS scores from day 1 to day 46 indicated that PANAS scores changed over time (time effect: F(2/122) = 2.7, p = 0.07, f = 0.21) and that groups differed in the long-term changes of positive and negative affect (time by group interaction effect: F(4/246) = 3.3, p = 0.01, f = 0.23), with group differences with regard to positive (F(2/122) = 2.8, p = 0.06, f = 0.21) and negative affect (F(2/122) = 4.7, p = 0.01, f = 0.28). Post-hoc univariate comparisons of PANAS scores on day 46 indicated that with regard to positive affect, interventions groups did not differ from each other, but both differed from the control group (causality group vs. control group: F(1/88) = 4.2, p = 0.04, f = 0.21; reversed causality group vs. control group: F(1/82) = 3.6, p = 0.06, f = 0.2; causality group vs. reversed causality group: F(1/68) = 0.02, p = 0.97). With regard to negative affect the reversed causality group did not differ from the control group, but both groups differed from the causality group (causality group vs. control group: F(1/88) = 6.3, p = 0.01, f = 0.27); reversed causality group vs. control group: F(1/82) = 0.5, p = 0.51; causality group vs. reversed causality group: F(1/68) = 9.6, p = 0.003, f = 0.37). Standardized mean differences for significant effects in the ITT sample were of small to medium size for positive affect (standardized between-groups differences at follow-up: causality group versus control group d = 0.41; reversed causality group versus control group d = 0.43; standardized within-group baseline to follow-up differences: causality group d = 0.46; reversed causality group d = 0.30;control group d = 0.01) and for negative affect (between groups at follow-up: causality group versus control group, d = 0.57; causality group versus reversed causality group, d = 0.64; standardized within group baseline to follow-up differences: causality group, d = 0.33; reversed causality group, d = -0.28; control group, d = -0.2).

## Discussion

The aim of this randomized-controlled trial was to test the feasibility of an open/hidden paradigm in psychotherapy research and to assess the effect of the assumingly incidental treatment constituent of providing a treatment rationale to the assumingly characteristic psychotherapeutic intervention of expressive writing.

First, both the intervention as well as the experimental manipulation proved to be valid and feasible, as indicated by the linguistic content analysis, the expected detrimental effects of expressive writing on affect during intervention days and participants rating of plausibility of their treatment rationale. Noteworthy, intervention groups did not differ in these parameters, so that it is unlikely that observed effects of the experimental manipulation of the treatment rationale are the consequence of differences in the perception, implementation and direct effects of the intervention itself. Second, while the experimental manipulation did not influence affect in short- and mid-term, we observed significant long-term differences between groups. Both intervention groups reported higher positive affect six weeks after the intervention in comparison to the no-treatment control group. In contrast, while the causality group showed a decrease of negative affect, the reversed causality group did not seem to benefit from the intervention in the same fashion. All of these group differences were of medium effect size and the differences between the reversed causality and causality group in negative affect at follow-up exceeded the magnitude of observed within group-changes between baseline and follow-up.

In the following, the observed effects will be discussed from an expressive writing, a theoretical and a methodological perspective. With regard to the former, the results of the causality group followed the expected direction and time course, with a temporally delayed and medium-sized improvement in affect (e.g. [28, 30, 31]). Also, immediate deterioration of affect with long-term improvement is proposed as the normal psychokinetic of expressive writing interventions [27] and follow-up duration has been found to be a significant predictor of expressive writing effects, with longer-term follow-up studies showing better outcome than studies with shorter-term follow-up [38]. It needs to be noted that although the observed effects are comparable to previous publications (see above), our trial is at best considered an efficacy trial and thus our results do not shed light on the effectiveness of online expressive writing interventions.

From a theoretical perspective, the observed response differences between the intervention groups are per se not unexpected. For example, a recent within-subject randomized blinded balanced placebo design observed higher analgesic effects with overt in contrast to covert lidocaine injections in healthy subjects [21]. Similarly, providing an otherwise similar aerobic exercise training or a work-related behavior with group-specific therapeutic rationales led to larger changes in self-esteem [24] or physiological health parameters [23], respectively. However, it needs to be noted that the reversed causality group benefitted from the intervention with regard to positive, but not to negative affect. Here, an early study on the effects of either open or hidden administration of a pharmacological intervention is informative. Ross and colleagues [39] observed discomfort in subjects receiving 10 mg of d-amphetamine covertly, while the overt administration resulted in neutral to comfortable mood, arguing that subjects who covertly received the pharmacological intervention, "had no 'therapeutic' set and presumably reported their feelings 'honestly' on the mood scales" (cited from [30], p. 391). However, the processes underlying the observed differences, such as differences in experiencing or attributing symptoms or both, need to be elucidated in further studies.

Regarding the methodological approach, the observed effects of our trial support the feasibility and utility of an open/hidden administration to disentangle effects of incidental treatment constituents from those of characteristic treatment constituents. In this regard, the beneficial effects on positive affect in both intervention groups could be seen as being caused by characteristic constituents of expressive writing, while the incidental treatment constituent of providing a treatment rationale is needed to obtain full effects, i.e. beneficial changes in both positive and negative affect. It needs to be noted that the definition of what is to be considered characteristic or incidental in psychotherapy is complicated by the fact that many treatments include the provision of a treatment rationale without defining its role in the treatment theory as characteristic. While this in general highlights the need to revise treatment theories [40, 41], the definition of what is to be considered incidental or characteristic in the case of expressive writing was considerably facilitated by the fact that the original instruction [42] did not contain the provision of a treatment rational and thus is clearly considered incidental to the treatment. According to Grünbaum's conceptualization [13], a treatment qualifies as verum, which he chooses to label "non-placebo", when at least one characteristic treatment constituent is therapeutic for a given disorder. With regard to our findings, we have reason to assume that expressive writing qualifies as verum, since its effects are observable even when administered covertly. However, there is an important amendment to this conclusion. Explanatory models and theories of expressive writing currently focus on emotion-related processes, i.e. propose disinhibition, emotional catharsis, cognitive-processing, self-regulation, exposure, social integration or development of a coherent narrative as underlying principles of change [27, 43]. Based on our results and given that 1) expressive writing effects are strongly associated with positive response expectancies, 2) a full confrontation with the traumatic experience is not a prerequisite for these effects [31] and 3) that writing about traumatic events is as beneficial as writing about positive events [43], future research is warranted to elucidate characteristic treatment constituents in expressive writing and to revise the underlying treatment theory accordingly.

There are several aspects of our study that need to be considered critically. First, participants in the reverse causality group may have known about the beneficial effects of writing. However, controlling for this is difficult, if not impossible, as asking participants about their assumptions regarding possibly positive effects of writing might itself provoke or prime such assumptions. Therefore, we opted against a direct inquiry and instead assessed the plausibility of the respective condition rationale. Our results showed no difference between the intervention groups, which indicates that participants found both possible causal directions between affect-change and writing equally acceptable.

Second, we experimentally manipulated the treatment rationale and thus provided the causality group, i.e. the open administration of the intervention, with a description of expected positive effects of the intervention. In the original instructions (e.g. [42]) this clear description and direction of effects is missing. However, the therapeutic properties of expressive writing interventions are inherently tied to accounts of this intervention (e.g. [44]) and even with the original instructions, i.e. without providing a description of expected positive effects, high positive expectancies that the writing intervention will have beneficial effects were found in comparable populations [30]. Also–at least with respect to ethical guidelines in psychotherapy–providing a treatment rational is integral to normative guidelines (see [45]). Since our aim was to test the effects of an experimental manipulation of providing a treatment rationale (and not to replicate the effects of the original expressive writing instructions), we choose to make the implicit explicit, by providing and manipulating the rationale of the two conditions. ‬‬‬‬‬‬‬‬However, it needs to be acknowledged that our instructions differed from those in Pennebaker [42], which did not provide a description of any anticipatory effect or treatment rationale.

Third and closely related to the previous aspect, our hidden administration of the online expressive writing intervention differs from similar approaches in medical research, where the patient is unaware when a given substance is administered. Instead, we operationalized the hidden administration by providing an alternative non-therapeutic rationale to ensure that both experimental conditions do not differ in their credibility and to minimize the possibility that the expressive writing intervention would per se be perceived as therapeutic. Given that this has been observed in trials not providing a rationale (e.g. [31]), we addressed this problem by implementing a non-therapeutic rationale.

Fourth, it needs to be noted that we carried out a large number of analyses and thus there is a probability that our main finding is due to chance. However, it needs to be considered that 1. we carefully analyzed pre-defined hypotheses with multivariate methods, 2. our finding of a significant short-term deterioration and a long-term improvement replicates previous findings on expressive writing from independent groups and 3. our main finding of a significant group difference in the course of long-term negative affect is of medium effect size and the consequence of a well-controlled experimental design. Therefore, we consider the probability of a chance finding as minimal. Finally, several of our finding in the ITT analyses failed to meet the 5% significance level (but fell under the 10% error level). While this could be taken as non-significance, we chose to consider these findings as of borderline significance, since the results in the ITT sample mirrored those in the per protocol sample.

To conclude, we consider the manipulation of assumingly incidental treatment constituents while keeping assumingly characteristic treatment constituents constant, as a promising design for psychotherapy research. Although our study might also illustrate the difficulties of this approach, it is far from new. Faced with the problem of an effective treatment without a proven mechanism, Benjamin Franklin and colleagues employed probably the first documented open/hidden trial to test Anton Mesmer’s animal magnetism at the end of the 18th. century. Using a specially designed blindfold, the servant of an experienced practitioner of magnetism was put to test: "Magnetized next with eyes uncovered, he feels tingling in his forehead when the metal rod is brought close to it; blindfolded again, he feels no tingling when the rod is brought close" (cited from [46], page 347). These results, amongst others, convinced the Franklin commission that "having finally demonstrated by decisive experiments that the imagination without magnetism produces convulsions, & that magnetism without imagination produces nothing" or state otherwise that the otherwise impressive effects of magnetism was fully driven by its incidental and not by any characteristic treatment constituents.

## Supporting information

S1 File CONSORT 2010 checklist. (DOC)

S2 File Instructions for experimental groups. (DOCX)

S3 File IRB trial protocol. (PDF)

S4 File Study protocol. (DOCX)

S5 File DRKS0000942. (PDF)

S6 File EW.csv. (CSV)
