# Supplementary material for: Employing open/hidden administration in psychotherapy research: A randomized-controlled trial of expressive writing
Source: PLoS One. 2017 Nov 27;12(11):e0187400. doi: 10.1371/journal.pone.0187400 (PMC5703461; doi:10.1371/journal.pone.0187400)
Supplement: S2 File — (DOCX) [file pone.0187400.s002.docx]

**Online Expressive Writing**

Instruction for expressive writing in causality and reversed causality groups (based on Horn & Mehl, 2004, S. 275, original in German, translation by authors)

Hello, my name is XX. Thank you very much for participating in this research study. *For the next 3 days, I would like for you to write about your very deepest thoughts and feelings concerning the most traumatic experience of your life. Alternatively, you can write about a very important emotional and personal issue that has affected your life profoundly. In your writing I would like you to really let go and explore your very deepest emotions and thoughts. You might relate your experience to your relationships with others, including parents, lovers, friends, or relatives. Also, you can relate your experience with your own history, your present and your future or who you have been then, who you would like to be or who you are now. You may write about the same general issue or experience on all days or on a different topic on each of these three days. All of your writing will be completely confidential. Do not worry about spelling, sentence structure, or grammar. The only rule is that once you begin writing, continue to do so until your time is up.*

Rationale of intervention and study aims for causality group (translation by authors, see also [video](http://youtu.be/BKGG2dGaV3A) in German)

*Research has shown that repeated writing about troubling traumatic experiences has a beneficial influence on psychological as well as somatic outcome. For example, numerous empirical studies have shown that writing about emotional problematic experiences in educational and clinical professions has an important influence on the emotional and somatic well-being. In 2004, Smith and Fahrenberger have proposed that so-called "Pondering Attention Shift Model (PASM)" to explain these effects, stating that by writing and reflecting about the traumatic experiences its apperception will change. In consequence, cognitive conflicts and emotional tensions will resolve, which in the long-run enhances subjective well-being. The goal of our study is both to replicate these improvements of well-being as well as examine possible causes of these beneficial effects and to further improve this effective intervention.*

Rationale of intervention and study aims for reversed causality group (translation by authors, see also [video](http://youtu.be/L94XQt5r6Ww) in German)

*Research has shown that how you feel has a profound influence on how things that happen to you are experienced, constructed and formulated. For example, numerous empirical studies have shown that weather conditions had a decisive impact on how employees were evaluated by their line managers. In 2004, Smith and Fahrenberger have proposed that so-called "Pondering Attention Shift Model (PASM)" to explain these effects, stating that the subjective well-being has a profound effect on the selective attentiveness. In consequence, the perception and representation of troubling experiences is determined by the respective current state of subjective well-being. The goal of our study is to examine how different aspects of the subjective well-being have an impact on how you write about traumatic experiences or put otherwise, how your feelings impact how you write and what mediates these effects.*
