# Supplementary material for: Employing open/hidden administration in psychotherapy research: A randomized-controlled trial of expressive writing
Source: PLoS One. 2017 Nov 27;12(11):e0187400. doi: 10.1371/journal.pone.0187400 (PMC5703461; doi:10.1371/journal.pone.0187400)
Supplement: S3 File — (PDF) [file pone.0187400.s003.pdf]

UNIVERSITÄT BASEL

FAKULTÄT FÜR PSYCHOLOGIE

Institutional Review Board
Dekanat
Missionsstrasse 62a
4055 Basel

English translation added in red

**Eingangsdatum:**		
**Antragsnummer:**		
**Versionsnummer:**		

Institutional Review Board
der Fakultät für Psychologie der Universität Basel

Antrag zur Durchführung von Untersuchungen mit freiwilligen Testpersonen

1.	Rahmenantrag:<br><br>Antrag auf Einzelstudie:	☐<br><br>☒
	Im Folgenden sind sowohl Einzelstudien als auch Rahmenanträge generisch benannt als „Untersuchung“.	
2.	Name des Antragstellers oder der Antragstellerin[1] mit Qualifikation (Master, Promotion, etc.), Abteilung, Kontaktadresse:<br><br>**Responsible / principal investigator**<br><br>Name des Projektleiters oder der Projektleiterin[2] mit Qualifikation (Promotion, etc.), Abteilung, Kontaktadresse:	Prof. Dr. Jens Gaab<br><br>Klinische Psychologie und Psychotherapie<br><br>s.o.

[1] Der/die Antragsteller/in muss mindestens über einen Masterabschluss verfügen
[2] Der/die Projektleiter/in muss mindestens über einen Promotionsabschluss verfügen

Institutional Review Board
Dekanat
Missionsstrasse 62a
4055 Basel

3.	Name der Untersuchung:<br>Title of study	EMPLOYING PLACEBO-DESIGNS IN PSYCHOTHERAPY RESEARCH: A RANDOM-IZED-CONTROLLED OPEN/HIDDEN EVALUATION OF EXPRESSIVE WRITING
4.	Begin of study<br>Geplanter Beginn der Untersuchung:	running since 2009 (at University of Zürich)<br>läuft seit 2009 (Beginn an der UZH)
5.	Scheduled termination of study<br>Geplantes Ende *der Datenerhebung*	31.12.2015
6.	Handelt es sich um eine Folgeuntersuchung zu einer bereits genehmigten Untersuchung?	
	**Wenn ja, legen Sie dem IRB bitte den ausgefüllten Fragebogen zur alten Untersuchung vor!**	
7.	Planned follow-up studies?<br>Sind Folgeuntersuchungen geplant?	no<br>nein
	**Bei Veränderungen der geplanten Untersuchung (z.B. Instrumente, Personengruppe, Design) ist ein neuer Antrag zu stellen.**	
8.	Kurzbeschreibung der Studie. Bitte stellen Sie das Untersuchungsziel, die Untersuchungsfrage und das Untersuchungsdesign kurz dar.<br>Abtract of study, including aims, methods and design	Psychotherapy has been shown to be effective, but efforts to prove specific effects by placebo-controlled trials have been practically and conceptually hampered. Here, we propose that adopting trial designs from placebo research with a reversed approach, i.e. to manipulate the incidental while con-trolling for characteristic treatment constituents instead of manipulating the characteristic while controlling for incidental treatment constituents, would offer a possible way to establish specificity in psychotherapy. Therefore, we test the effects of providing opposing treatment rationales to an identical psychotherapy intervention (online expressive writing) in a randomized-controlled open/hidden administration trial on positive and negative affect in healthy subjects.
9.	Auf welchem Weg (z.B. über das Internet, mithilfe einer Organisation) rekrutieren Sie Ihre Untersuchungsteilnehmer? Falls Sie das Internet nutzen, geben Sie bitte auch an, wer Ihre Adressaten sind bzw. wie Sie Ihre Teilnehmer „finden".	ja, Studierende im BSc-Studiengang<br>Online, bachelor students

Recruitment: Approach and population

Institutional Review Board
Dekanat
Missionsstrasse 62a
4055 Basel

10.	Angaben zu den Studienteilnehmern (Anzahl, Altersbereiche, Geschlecht):<br>Participants: N, age and gender	angestrebter N=180, gesunde Männer und Frauen ab 18 Jahren<br>Participants: N=180, 18 years and older and all gender
	Besondere Auswahlkriterien bei den Untersuchungsteilnehmern (etwa soziale oder ethnische Herkunft, Krankheiten, Behinderungen, Leistungen, andere Merkmale):	-
11.	Die Untersuchung schliesst ein:<br>Study involves:	☐ keine Manipulation oder Täuschung der Teilnehmer und keine Risiken für sie<br>☐ manipulative Fragen<br>☐ verdeckte Beobachtung<br>☒ Täuschungen der untersuchten Person<br>deception<br>☐ physische Risiken<br>☐ psychische Risiken
	**Falls Sie die Ziele Ihrer Untersuchung nur mithilfe manipulativer Fragen, verdeckter Beobachtungen oder Täuschungen erreichen können, sind die Untersuchungsteilnehmer nachträglich aufzuklären und über Sinn und Zweck der Untersuchung zu informieren.**	
12.	If participants are deceived, will they be informed after termination of trial?<br>Wie werden Sie die Probanden im Anschluss an die Untersuchung aufklären, falls Ihre Untersuchung manipulative Fragen, verdeckte Beobachtung oder Täuschungen einschliesst?	Ja<br>yes
13.	Falls Ihre Untersuchung physische oder psychische Risiken für die Untersuchungsteilnehmer einschliesst: Wie wollen Sie mögliche Risiken verringern?<br>Are there any risks for participants?	keine Risiken<br>no risks
	**Die Teilnehmer sind vor Beginn der Untersuchung über die Risiken aufzuklären und müssen durch ihre Unterschrift bestätigen, dass sie mit den Untersuchungsbedingungen einverstanden sind.**	
14.	Werden individuumsbezogene sensible Daten (wie z.B. Sexualverhalten, Drogenmissbrauch, finanzielle Situation) erhoben? Wenn ja, bitte angeben welche:<br>Do you assess any senisble data (e.g. sexual behavior, drug use, financial issues)?	nein<br>no
	**Im Fall der Erhebung sensibler Daten sind die Teilnehmer vor Beginn der Untersuchung darüber zu informieren.**	

UNI
BASEL

Institutional Review Board
Dekanat
Missionsstrasse 62a
4055 Basel

15.	Von den Probanden werden Angaben zu folgenden Aspekten erhoben:<br><br>Assessmen of participant characteristics	☒ Geschlecht Gender<br>☒ Alter Age<br>☐ Ethnische Herkunft<br>☐ Familienstand<br>☐ Einkommen<br>☐ AHV-Nummer<br>☐ Adresse<br>☐ Telefonnummer
16.	Untersuchungsinstrumente:<br><br>Methods	☒ Standarduntersuchungsinstrumente (z.B. bestehende Fragebögen oder Computer-aufgaben) exisiting methods (questionnaires, computer tasks, etc)<br><br>☐ im Projekt entwickelte Untersuchungs-instrumente
	Bitte legen Sie dem IRB die Untersuchungsinstrumente vor, die Sie in Ihrem Projekt verwenden wollen, bzw. geben Sie dem IRB Gelegenheit, Ihre Instrumente oder Verfahren kennen zu lernen (u.a. Angabe von Referenzen bei Standardverfahren). Bei Rahmenanträgen sollen konzeptuell und prozedural-methodisch ähnliche Untersuchungsinstrumente anhand eines Beispiels illustriert werden.	
17.	Datenspeicherung:<br>data storage	☐ Aufzeichnungen auf Papier<br>☒ Aufzeichnungen auf elektronische Datenträger digital<br>☐ Aufzeichnungen mithilfe audio-visueller Medien
	Die Teilnehmer sind vor Beginn der Untersuchung über Art und Dauer der Speicherung und Weiterverwendung von personenbezogenen audiovisuellen Aufzeichnungen aufzuklären, falls diese nicht anonymisiert aufbewahrt werden. Die Teilnehmer müssen durch ihre Unterschrift bestätigen, dass sie mit den Untersuchungsbedingungen einverstanden sind.	
18.	Werden Daten ganz oder teilweise von einem externen Dienstleister erhoben, gespeichert oder verarbeitet?<br>Will any data be handled or stored by external providers?	☐ Ja<br>☒ Nein No<br><br>Wenn ja, bitte erläutern Sie, wie Sie die Anonymität und Wahrung der Persönlich-keitsrechte der Untersuchungsteilnehmer/innen gewährleisten wollen.

Institutional Review Board
Dekanat
Missionsstrasse 62a
4055 Basel

	Die Teilnehmer sind vor Beginn der Untersuchung über Art und Dauer der Speicherung und Weiterverwendung von personenbezogenen audio-visuellen Aufzeichnungen aufzuklären, falls diese nicht anonymisiert aufbewahrt werden. Die Teilnehmer müssen durch ihre Unterschrift bestätigen, dass sie mit den Untersuchungsbedingungen einverstanden sind.	
19.	Wie werden die Daten, mit denen Sie arbeiten, anonymisiert? Anomymization of data?	ja Yes
20.	Wie werden die Originaldaten Ihrer Untersuchungsteilnehmer (z.B. ausgefüllte Fragebögen, Testhefte, Adressen, Einwilligungserklärungen, Bankverbindungen) und die Daten zur Entschlüsselung von Codes aufbewahrt? Separation of personal data and identifyer/coding?	separates File, welches nur dem VL zugänglich ist<br>Yes, separate files, only accessible to PI
21.	Erhalten Ihre Untersuchungsteilnehmer eine Aufwandsentschädigung (z.B. monetär, Kreditpunkte als "Unterschrift")? Reimbursement	Kreditpunkte<br>Study credits, needed for BSc studies
	Wie wird eine potentielle Bezahlung geregelt, wenn die Teilnahme an der Untersuchung vorzeitig abgebrochen wird? Reimbursement also in case of termination before study completion?	Kreditpunkte entsprechend Aufwand<br>Yes, then study credits according to time spent in trial
	Die Untersuchungsteilnehmer sind vor Beginn der Untersuchung über die Höhe der Aufwandsentschädigung zu informieren.	

UNI BASEL
