# Supplementary material for: Employing open/hidden administration in psychotherapy research: A randomized-controlled trial of expressive writing
Source: PLoS One. 2017 Nov 27;12(11):e0187400. doi: 10.1371/journal.pone.0187400 (PMC5703461; doi:10.1371/journal.pone.0187400)
Supplement: S4 File — (DOCX) [file pone.0187400.s004.docx]

**Antragspunkt 8 – Kurzbeschreibung der Studie**

*Dieses Formular ist dem „Antrag zur Durchführung von Untersuchungen mit freiwilligen Testpersonen“ beizulegen!*

Name des Projektleiters / der Projektleiterin^[[1]](#footnote-1)^: Jens Gaab

Name des Projekts^[[2]](#footnote-2)^: EMPLOYING PLACEBO-DESIGNS IN PSYCHOTHERAPY RESEARCH: A RANDOM-IZED-CONTROLLED OPEN/HIDDEN EVALUATION OF EXPRESSIVE WRITING

| **Untersuchungsziel**  (Allgemeine Ziele der Studie) | Psychotherapy has been shown to be effective, but efforts to prove specific effects by placebo-controlled trials have been practically and conceptually hampered. Here, we propose that adopting trial designs from placebo research with a reversed approach, i.e. to manipulate the incidental while controlling for characteristic treatment constituents instead of manipulating the characteristic while controlling for incidental treatment constituents, would offer a possible way to establish specificity in psychotherapy. |
| --- | --- |
| **Untersuchungsfrage**  (Genauere Hypothesen) | We tested the effects of providing opposing treatment rationales to an identical psychotherapy intervention (online expressive writing) in a randomized-controlled open/hidden administration trial on positive and negative affect in healthy subjects. |
| **Untersuchungsdesign**  (Typ von Studie, Studienablauf inkl. Zeitangaben, verwen­dete Mess­grössen, primäre und sekundäre Zielvariablen etc.) | see next pages |

Untersuchungsdesign

We conduct a randomized controlled trial with three experimental conditions in healthy subjects. Two groups undergo a standardized online intervention, whereas the control group does not receive an intervention. While both intervention groups receive the same online intervention, they are given different treatment rational for the intervention: While participants in the causality group are told that the intervention will in the long-run have beneficial effects on mood in the long-run, participants in the reversed causality group are instructed that mood will influence how they will perform in the intervention (see Appendix for details).

As intervention, we employ an online version of the expressive writing paradigm (Pennebaker & Beall, 1986), because of its feasibility and proven efficacy to reduce distress in healthy student and clinical populations (e.g. Baikie, Geerligs, & Wilhelm, 2012; Beyer et al., 2014; Halpert, Rybin, & Doros, 2010; Schutte, Searle, Meade, & Dark, 2012).

In total, participants are imbedded in the study for 46 days. The study encompass three intervention days (days 1 to 3) and four assessments (days 1, 4, 10 and 46 or baseline, post intervention, mid-term and long-term follow-up, respectively). On intervention and assessment days participants receive an email with an individual access code for a webpage differing in content according to group assignment and study day. For intervention groups, the website contains the outcome (PANAS, see below) and on intervention days an embedded video, showing a professional speaker explaining the rationale of the intervention. While the instruction is equal for both intervention groups in terms of structure and format, the treatment rationale, i.e. proposed meaning, differed in content between these groups (see Appendix for details). For the control group, this website contains the outcome only. On intervention days, participants of the intervention groups complet the outcome before and after the expressive writing intervention, while the control group complet the outcome only once on each of the intervention days. The intervention, the different instructions as well as the assessments are conducted online in order to control for patient-therapist interaction. Participants are not informed about the existence of and the difference between the three groups. Subjects will be debriefed after study participation.

Measures

Possible effects of the experimental conditions are repeatedly assessed for up to 46 days after baseline with the Positive and Negative Affect Schedule (PANAS, Krohne, Egloff, Kohlmann, & Tausch, 1996; Watson, Clark, & Tellegen, 1988), which is a priori defined as outcome. The PANAS contains two scales with overall 10 five-point items assessing positive (e.g. "interested", "proud") and negative affect (e.g. "upset", "ashamed"). In the intervention groups, linguistic content of written text, subjective rating of the severity of the reported traumatic experiences and the plausibility of their respective treatment rationale are employed to assess the validity of the intervention and experimental manipulation of treatment rational. The linguistic content of the written text was analyzed with Linguistic Inquiry and Word Count (Pennebaker, Francis, & Booth, 2001; Wolf et al., 2008), which relia-bly quantifies words in a given text according to preset categories. For the purposes of this study, we use the word count of first person singular personal pronouns (e.g. I, me, mine), of negative and positive emotions (e.g. sad, hate, worthless and happy, pretty, good), of cognitive processes (e.g. distinguish, because, know), of causality (e.g. argument, influence, effect) and of insight (e.g. recognize, conscious, decision). Subjective rating of the severity of the traumatic experiences is assessed with a single sentence 5-point item (i.e. In general, how distressing is the experience you have just written about for you?) and plausibility of the treatment rationale is operationalized with one 5-point item for each group (causality group: Writing about a traumatic experience influences my well-being, re-versed causality group: My well-being influences how I write about a traumatic experience).

Subjects

Subjects are recruited amongst psychology students at University of Basel through mailing lists and web postings at both universities. Inclusion criteria are (1) ages of 18 years and older, (2) absence of any mental disorder by self report, (3) not receiving psychological or psychiatric or medical treatment in the last six month by self-report and (4) a Toronto Alexithymia Scale score below 54 (Kupfer, Brosig, & Brahler, 2000), since alexithymia has been shown to influence effects of expressive writing (Paez, Velasco, & Gonzalez, 1999; Solano, Donati, Pecci, Persichetti, & Colaci, 2003).

Upon eligibility and after signing the informed consent, participants complete the Toronto Alexithymia Scale, the PANAS as well as provided descriptive and demographic information.

Statistics

Based on assumed small to medium effects of our experimental manipulation of treatment rationale on intervention effects, a priori sample size calculation with the statistical software G*Power 3.1 (Faul, Erdfelder, Buchner, & Lang, 2009) leads to an optimal sample size of N = 120 (f= 0.2, 80% power, 5% alpha error, 3 groups, 4 assessments and correlation among repeated measures = 0.3). We assume that our restrictive exclusion criteria, the population under investigation and the emotionally demanding task would lead to a substantial dropout of at least 30%. We therefore set out to recruit at least N=180. SPSS 21 statistical software (SPSS, Chicago, Illinois, USA) for Apple OS X will be used for all statistical analyses. Analysis of variance or chi-square tests are to be used to examine demographic and clinical variables at baseline. A time by group by scale multivariate analysis of variance with subsequent time by group univariate analysis of variance for single PANAS scales are to be used to investigate differences between groups regarding treatment effects over time. For significant results between groups, Cohens f (calculated from SPSS partial eta-squared with the program G*Power 3.1: 0.1=small, 0.25=medium and 0.45=large) are to be used for time by group interaction effects. Within- and between-group effect sizes were calculated using Cohen's d (0.2=small, 0.5=medium, 0.8=large). All results are displayed as mean values and standard deviation unless otherwise indicated. All analyses are per-formed on an intent-to-treat (ITT) basis including randomized patients with both baseline and at least post-intervention value (day 4) for the variable being analyzed.

1. Gemäss Ziffer 2 des Antragsformulars [↑](#footnote-ref-1)
2. Gemäss Ziffer 3 des Antragsformulars [↑](#footnote-ref-2)
