# Supplementary material for: Employing open/hidden administration in psychotherapy research: A randomized-controlled trial of expressive writing
Source: PLoS One. 2017 Nov 27;12(11):e0187400. doi: 10.1371/journal.pone.0187400 (PMC5703461; doi:10.1371/journal.pone.0187400)
Supplement: S5 File — (PDF) [file pone.0187400.s005.pdf]

PLEASE NOTE: *This trial has been registered retrospectively.*

Trial Description

Title

Employing place- bo-designs in psychotherapy research: A randomized-controlled open/hidden evaluation expressive writing

Trial Acronym

EW

URL of the trial

[---]*

Brief Summary in Lay Language

Employing an online expressive writing intervention, we tested the feasibility of an open/hidden paradigm in psychotherapy and whether the effects of the assumingly characteristic treatment constituent of expressing emotions about a traumatic experience are influenced by the assumingly incidental treatment constituent of providing a therapeutic rationale.

Brief Summary in Scientific Language

Psychotherapy has been shown to be effective, but efforts to prove specific effects by placebo-controlled trials have been practically and conceptually hampered. Here, we propose that adopting open/hidden designs from placebo research would offer a possible way to establish specificity in psy-chotherapy. Therefore, we tested the effects of providing opposing treatment rationales in an online expressive writing intervention on affect in healthy subjects. Results show that it was possible to con-duct the expressive writing intervention both covertly and openly, but that participants in the hidden administration condition did not fully benefit from the otherwise effective expressive writing interven-tion in the long-run. Noteworthy, effect sizes between open and hidden administration groups were comparable to pre-post effect sizes of the intervention. While this finding is important for the under-standing of psychotherapy's effects per se, it also proves that alternative research approaches to estab-lish specificity are feasible and informative in psychotherapy research.

Organizational Data

- DRKS-ID: **DRKS00009428**
- Date of Registration in DRKS: **2015/09/29**
- Date of Registration in Partner Registry or other Primary Registry: **[---]***
- Investigator Sponsored/Initiated Trial (IST/IIT): **yes**
- Ethics Approval/Approval of the Ethics Committee: **No approval required according to EC**
- (leading) Ethics Committee Nr.: **/ , Kantonalen Ethikkommission**
  Kanton Zürich
  Schweiz

Secondary IDs

Health condition or Problem studied

- Free text: **Healthy subjects, i.e. without any somatic or psychiatric acute or chronic disease or disorder by self-report**

Interventions/Observational Groups

- Arm 1: **Expressive writing intervention (writing for 30 min each on three consecutive days about a distressing experience). Particiants were informed that writing about a distresssing experience in the midrun improves mood.**
- Arm 2: **Expressive writing intervention (writing for 30 min each on three consecutive days about a distressing experience). Particiants were informed that mood had an effect on writing about a distresssing experience.**
- Arm 3: **The control group received no treatment**

Characteristics

- Study Type: **Interventional**
- Study Type Non-Interventional: **[---]***
- Allocation: **Randomized controlled trial**
- Blinding: **[---]***
- Who is blinded: **patient/subject**
- Control: **Active control (effective treament of control group), Control group receives no treatment**
- Purpose: **Treatment**
- Assignment: **Parallel**
- Phase: **N/A**
- Off-label use (Zulassungsüberschreitende Anwendung eines Arzneimittels): **N/A**

Primary Outcome

Possible effects of the experimental conditions were repeatedly assessed for up to 46 days after base-line (baseline=Tag 1, post-Expressive Writing=Tag 4, midterm follow up=Tag 10 und longterm-follow-up=Tag46) with the Positive and Negative Affect Schedule (PANAS, Krohne, Egloff, Kohlmann, & Tausch, 1996), which was a priori defined as outcome. The PANAS contains two scales with overall 10 five-point items assessing positive (e.g. "interested", "proud") and negative affect (e.g. "upset", "ashamed").

Secondary Outcome

/

Countries of recruitment

- CH **Switzerland**

Locations of Recruitment

- other **Fakultät für Psychologie, Universität Basel, Basel**

Recruitment

- Planned/Actual: **Actual**
- (Anticipated or Actual) Date of First Enrollment: **2011/10/01**
- Target Sample Size: **120**
-

Planned/Actual: **Actual**

(Anticipated or Actual) Date of First Enrollment: **2011/10/01**

Target Sample Size: **120**

Monocenter/Multicenter trial: **Monocenter trial**

- National/International: **National**

Inclusion Criteria

- Gender: **Both, male and female**
- Minimum Age: **18 Years**
- Maximum Age: **no maximum age**

Additional Inclusion Criteria

**(1) ages of 18 years and older,
(2) absence of acute or chronic mental disorder or somatic disease by self report,
(3) not receiving psychological or psychiatric or medical treatment in the last six month by self-report and
(4) a Toronto Alexithymia Scale score below 54 (Kupfer, Brosig, & Brahler, 2000), since alexithymia has been shown to influence effects of expressive writing (Paez, Velasco, & Gonzalez, 1999).**

Exclusion criteria

**(1) younger than 18 years
(2) any acute or chronic mental disorder or somatic disorder by self report
(3) receiving psychological or psychiatric or medical treatment in the last six month by self-report
(4) a Toronto Alexithymia Scale score above 54 (Kupfer, Brosig, & Brahler, 2000), since alexithymia has been shown to influence effects of expressive writing (Paez, Velasco, & Gonzalez, 1999).**

Addresses

- **Primary Sponsor**

Klinische Psychologie und PsychotherapieFakultät für PsychologieUniversität Basel

Mr. Prof. Dr. Jens Gaab
Missionsstrasse 62-64
4055 Basel
Switzerland

Telephone: **0041612670748**
Fax: **[---]***
E-mail: **jens.gaab at unibas.ch**
URL: **[---]***

- **Contact for Scientific Queries**

Klinische Psychologie und Psychotherapie Fakultät für PsychologieUniversität Basel

Mr. Prof. Dr. Jens Gaab
Missionsstrasse 62-64
4055 Basel
Switzerland

Telephone: **0041612670748**
Fax: **[---]***
E-mail: **jens.gaab at unibas.ch**
URL: **[---]***

- **Contact for Public Queries**

Klinische Psychologie und PsychotherapieFakultät für PsychologieUniversität Basel

Mr. Prof. Dr. Jens Gaab
Missionsstrasse 62-64
4055 Basel
Switzerland

Telephone: **0041612670748**
Fax: **[---]***
E-mail: **jens.gaab at unibas.ch**
URL: **[---]***

Sources of Monetary or Material Support

- **Institutional budget, no external funding (budget of sponsor/PI)**

  Klinische Psychologie und Psychotherapie Fakultät für PsychologieUniversität Basel

  4055 Basel
  Switzerland

  Telephone: **[---]***
  Fax: **[---]***
  E-mail: **[---]***
  URL: **[---]***

Status

- Recruitment Status: **Recruiting complete, follow-up complete**
- Study Closing (LPLV): **2014/10/01**

Trial Publications, Results and other documents

** This entry means the parameter is not applicable or has not been set.*
